# Replies to Queries

**Published:** 1873-06

**Authors:** 


*Replies to Queries.*

Dr. M. A. J.—Will please pardon the Senior Editor for declining to publish his article. The profession of Georgia need no such vindication. As a body, they are as true to principle as the "needle is to the pole."

Dr. T. W. G.—We are not able to answer your questions. We believe your friends are correct: "Respectability, supported alone by trickery and policy, will be of short duration." The best way to get rid of such characters is to give them plenty of rope, and let them, with the aid of their friends, hang themselves. No man who deserves honor will buy it, or plan and scheme to secure it. True honor is a gratuitous premium on merit.

Dr. E. L.—You need have no fears in regard to the integrity of the Record. It has never knowingly made a misstatement, and never will. Those who know us will indorse for our standing to what we believe to be the truth. We are satisfied with our character for *tenacity* to the truth, and contented with our faith in its ultimate success.

Dr. L. C. H.—In all ages we have had Ransey Sniffles and policy men, who would advocate every side of every question to advance their interest, but, like many of our manipulated fertilizers, soon pass away, and leave the world poorer than they found it.

Dr. C. B. P.—Say to your friends we will with pleasure, in the July number, republish the short article on medical colleges.

Dr. B. R.—We thank you for your long letter, and will endeavor to profit by your very kind suggestions and good advice. It will continue to be our purpose to make the Record a true exponent of our profession—a medium through which they can reach each other, and thereby advance the science of medicine. P.

